# Radiotherapy for small cell carcinoma of the esophagus: outcomes and prognostic factors from a retrospective study

**DOI:** 10.1186/s13014-019-1415-9

**Published:** 2019-11-21

**Authors:** Baoqing Chen, Han Yang, Huali Ma, Qiaoqiao Li, Bo Qiu, Yonghong Hu, Yujia Zhu

**Affiliations:** 1 Department of Radiation Oncology, State Key Laboratory of Oncology in South China, Collaborative Innovation Center of Cancer Medicine, Sun Yat-sen University Cancer Center, Guangzhou, 510060, P. R. China; 2 Guangdong Esophageal Cancer Institute, Guangzhou, P. R. China; 3 Department of Thoracic Surgery, State Key Laboratory of Oncology in South China, Collaborative Innovation Center of Cancer Medicine, Sun Yat-sen University Cancer Center, Guangzhou, 510060, P. R. China; 4 Department of Radiology, State Key Laboratory of Oncology in South China, Collaborative Innovation Center of Cancer Medicine, Sun Yat-sen University Cancer Center, Guangzhou, 510060, P. R. China

**Keywords:** Small cell carcinoma of esophagus, Radiation therapy, Chemotherapy, IMRT, Prognosis

## Abstract

**Background:**

Small cell carcinoma of the esophagus (SCCE) is characterized by its progressive feature and poor prognosis. There is no consensus on a standard therapeutic modality for SCCE. In this study, we aimed to characterize the outcomes of primary SCCE patients treated by radiation therapy as part of treatment and investigate prognostic factors.

**Methods:**

We retrospectively analyzed the data of 42 SCCE patients who were treated by RT as part of treatment at the Sun Yat-sen University Cancer Center from 2001 to 2014. The Kaplan-Meier and log-rank method were used to analyze survival. Cox’s hazard regression model was applied to determine prognostic factors.

**Results:**

Of the 42 enrolled patients, 25 had limited disease (LD) and 17 with extensive disease (ED). The overall response rate (CR + PR) was 60.0% (21/35). The median overall survival time (OS) for whole and LD group were 12.9 and 36.8 months. The 1-, 3- and 5-year OS rates of the whole cohort were 64.9, 31.3, and 13.9%, respectively. OS was significantly longer in patients with ECOG performance score (ECOG PS) < 2 (*p =* 0.001), lesion length ≤ 5 cm (*p* *=* 0.001), and LD (*p* = 0.049). In the patients with LD, multivariate analysis indicated that combined with chemotherapy (*P* = 0.046) and higher radiation dose (*P* = 0.027) predicted better prognosis in OS. The overall rate of grade 3–4 toxicities in the whole cohort was 37.5%. In total, 65% (17/26) patients with recurrent disease died with the metastasis with or without the primary recurrence.

**Conclusion:**

RT was one of the effective and safe treatments for locoregional control of SCCE. Lower ECOG PS score, shorter lesion length, treated with chemotherapy, and a higher dose of RT were identified as favorable independent prognostic factors.

## Introduction

Small-cell carcinoma is one kind of highly malignant neoplasm that usually originates from the lung. The first case of primary small cell carcinoma of the esophagus (SCCE) was reported by Mckeown in 1952 [1]. SCCE is characterized by its aggressive feature with poor prognosis and is distinct from the squamous cell carcinoma or adenocarcinoma of the esophagus, but similar to small cell carcinoma arising from lungs or other organs. A consensus on the standard therapeutic modality for SCCE has not been established, but based on retrospective studies, a multimodal therapeutic approach combining surgery, radiotherapy (RT), and chemotherapy is recommended [2, 3]. When combined with chemotherapy, RT is considered a major local therapy approach for treating patients with localized or locoregional disease. Though RT is common in clinical practice in western countries, esophagectomy is more widely used compared to RT as the treatment for localized SCCE in China [2, 4]. A previous retrospective study of all the SCCE cases in our center from September 1990 to June 2011 showed that of 64 SCCE patients, only 15 (26.7%) patients received RT [5]. Due to increasing evidence showing its non-inferiority to esophagectomy, RT technology was developed over the years in China for treating SCCE. In this retrospective study, we focused on SCCE cases who had received RT and explored their outcomes and prognostic factors.

## Patients and methods

### Patients

We retrospectively reviewed 9547 cases of esophagus carcinoma at Sun Yat-sen University Cancer Center from June 2001 to December 2014. Among these cases, 110 (1.15%) patients were pathologically diagnosed as SCCE. Of these 110 patients, 45 (40.9%) had received RT as part of treatment. The inclusion criteria were as follows: 1) age between 18 and 75 years; 2) Eastern Cooperative Oncology Group (ECOG) performance status (PS) score ≤ 3; 3) received RT as part of the treatment; and 4) absence of previous thoracic RT. Finally, 42 patients with complete medical records were enrolled for analysis in this retrospective study.

Medical records retrieved were examined for medical history and physical examination; complete blood count and serum chemistry profile; electrocardiogram; barium-swallow examination; contrast-enhanced computed tomography (CT) scan of the neck, chest and upper abdomen, endoscopic ultrasound, ultrasound of the cervical lymph node, PET-CT, radioactive isotope bone scans, if available. The disease stage was presented as either a limited-stage disease (LD) or an extensive-stage disease (ED) according to the Veteran’s Administration Lung Group’s 2-stage classification scheme (VALSG) for primary pulmonary small cell lung cancer (SCLC) [6]. LD is defined as a tumor confined within a localized anatomical region, which can be safely encompassed within a radiation field and ED is defined as a tumor outside of the local regional region. Tumor location is defined based on the UICC 1987 standard.

The Ethics Committee of Sun Yat-sen University Cancer Center reviewed and approved this study, which was performed according to the principles of the Helsinki Declaration. Informed consent was obtained from each patient for the collection of clinical information at the first visit.

### Treatment

For RT administration, a vacuum cradle for immobilization was made with the patient in a supine position. Patients were scanned from the first cervical vertebra (C1) to the third lumbar vertebra (L3) level. CT scan was performed with 5 mm thick slices. The gross tumor volume (GTV) consisted of tumor shown by CT scans or PET/CT or endoscopy. Lymph nodes were defined as positive nodes if it exhibited any of the following features on CT: short axis ≥ 10 mm, distribution in a cluster of lymph nodes, infiltrative margin, or central necrosis. Lymph nodes that demonstrated high uptake on the PET/CT scan were also included in the GTV, regardless of size. The clinical target volume (CTV) comprised the original tumor or anastomosis site, supraclavicular, and station 1–5 and 7 lymph nodes. The plan target volume (PTV) 1 was defined as the GTV plus a margin of 5 mm and PTV2 was defined as the CTV plus a margin of 5 mm in all directions, respectively. Three-Dimensional Conformal Radiation Therapy (3D-CRT) treatment plans were calculated by Pinnacle planning system and Intensity Modulation Radiated Therapy (IMRT) treatment plans were calculated by the Monacle planning system. All patients were treated with a 6-MV linear accelerator. The prescribed dose was generally 50–66 Gy for PTV1 and 42–50 Gy for PTV2. Dose constraints for critical organs were spinal cord < 45 Gy; mean lung dose< 17 Gy and lung dose V20 < 30%.

### Statistics

Tumor response was assessed using Response Evaluation Criteria in Solid Tumors 1.1 (RECIST 1.1). The Overall Survival (OS) time of patients was calculated from the start of initial treatment to the date of death or last follow-up and calculated and compared using the Kaplan–Meier method and the log-rank p test. Multivariate analysis was performed using Cox regression to analyze the prognostic factors. *P* value< 0.05 was considered significant. All statistical analysis was performed using SPSS 22.0 software.

## Results

### Patient demographic and baseline characteristics

Clinical data including patient demographics and tumor characteristics are outlined in Table 1. The median age of patients was 55 years (range: 42–72 years) with 29 (69%) male and 13 (31%) female patients. Almost half (20/47.6%) patients had a history of heavy smoking (smoking index ≥400) or risky diets, which include heavy drinking, hot food or salt-preserved foods. Twenty-nine (69.0%) patients had an Eastern Cooperative Oncology Group (ECOG) performance status (PS) of 0–1, while 12 (31.0%) patients had an ECOG PS of 2–3. Middle thoracic SCCE was observed in 21(50.0%) patients and two cases had multi-origins SCCE. Histological analysis showed 39 (92.9%) patients with only small cell carcinoma whereas two patients had small cell carcinoma coexisting with squamous cell carcinoma, and another one with cardiac adenocarcinoma as well. At the time of diagnosis, 25 (59.5%) had LD and the other 17 (40.5%) patients had ED. Brain metastasis was not detected at the time of diagnosis but developed in two patients during follow-up.

**Table 1:** Patient information and tumor characteristics

	No.	Percentage %
Sex		
Male	29	69.0
Female	13	31.0
Age (55; range: 42–72 years)		
< 60 years	24	57.1
≥ 60 years	18	42.9
Smoking history		
yes	20	47.6
no	22	52.4
Risky diet		
yes	20	47.6
no	22	52.4
ECOG PS		
0–1	29	69.0
2–3	13	31.0
Location		
cervical	0	0
upper thoracic	10	20.8
middle thoracic	21	50.0
lower thoracic	9	21.4
multiple	2	4.8
Length (cm)		
≤ 5	20	47.6
> 5	22	52.4
Stage		
extensive-disease	17	40.5
limited-disease	25	59.5
Pathology		
Pure SCCE	39	92.9
Coexisting with SCC	2	4.8
Coexisting with Adenocarcinoma	1	2.4
Radiation technology		
3DCRT	24	57.1
IMRT	18	42.9
Radiation Dose(58; Range: 42-66Gy)		
< 56Gy	15	35.7
≥ 56Gy	27	64.3

Abbreviations: *ECOG PS*: the Eastern Cooperative Oncology Group Performance Status Scale, *SCCE*: Small Cell Carcinoma of esophagus, *SCC*:squamous cell carcinoma, *3DCRT*: 3-Dimensional Conformal Radiation Therapy, *IMRT*: Intensity Modulation Radiated Therapy

### Treatment

Of the 42 patients who received RT, 24 (57.1%) received 3DCRT treatment, and the remaining 18 (42.9%) patients received IMRT treatment. The median RT dose was 58 Gy that ranges from 42 to 66 Gy. The treatment regimens for patients with LD or ED are listed in Table 2. In the 25 patients with LD, seven patients received RT only, 13 patients were treated with RT combined with chemotherapy, while the left five received adjuvant RT ± chemotherapy after surgery. Prophylactic brain radiation at a dose of 30 Gy in 10 fractions was performed in two patients with LD. Of the 17 patients with ED, eight patients received RT alone, five patients were treated with RT combined with chemotherapy, and the left four patients received RT with chemotherapy and surgery (two received adjuvant RT after esophagectomy, and two received pre-operation RT).

**Table 2:** Treatment regimen of 42 SCCE patients

Treatment group	No.		
	LD	ED	All(%)
R	7	8	15 (35.7%)
R + C	13	5	18 (42.9%)
S + R ± C	5	4*	9 (21.4%)
Total	25	17	42

Abbreviations: *R*: radiation therapy; *C*: chemotherapy; *S*: surgery; *LD*: limited disease; *ED*: extensive disease;

*two received adjuvant RT after esophagectomy, and two received pre-operation RT

### Tumor response rate and survival

Tumor response was assessed at two to 3 months after the completion of treatment. Among those 35 patients who are eligible for response evaluation (seven patients with adjuvant therapy after surgery were excluded), five patients achieved complete remission (CR) and 16 achieved partial remission (PR). The overall response rate (CR + PR) was 60.0% (21/35). Eight patients achieved stable disease (SD), and six achieved progressive disease (PD). The overall disease control rate is 83%(29/35). There was no evidence of relapse in seven patients who received adjuvant RT after esophagectomy.

**Fig. 1:**
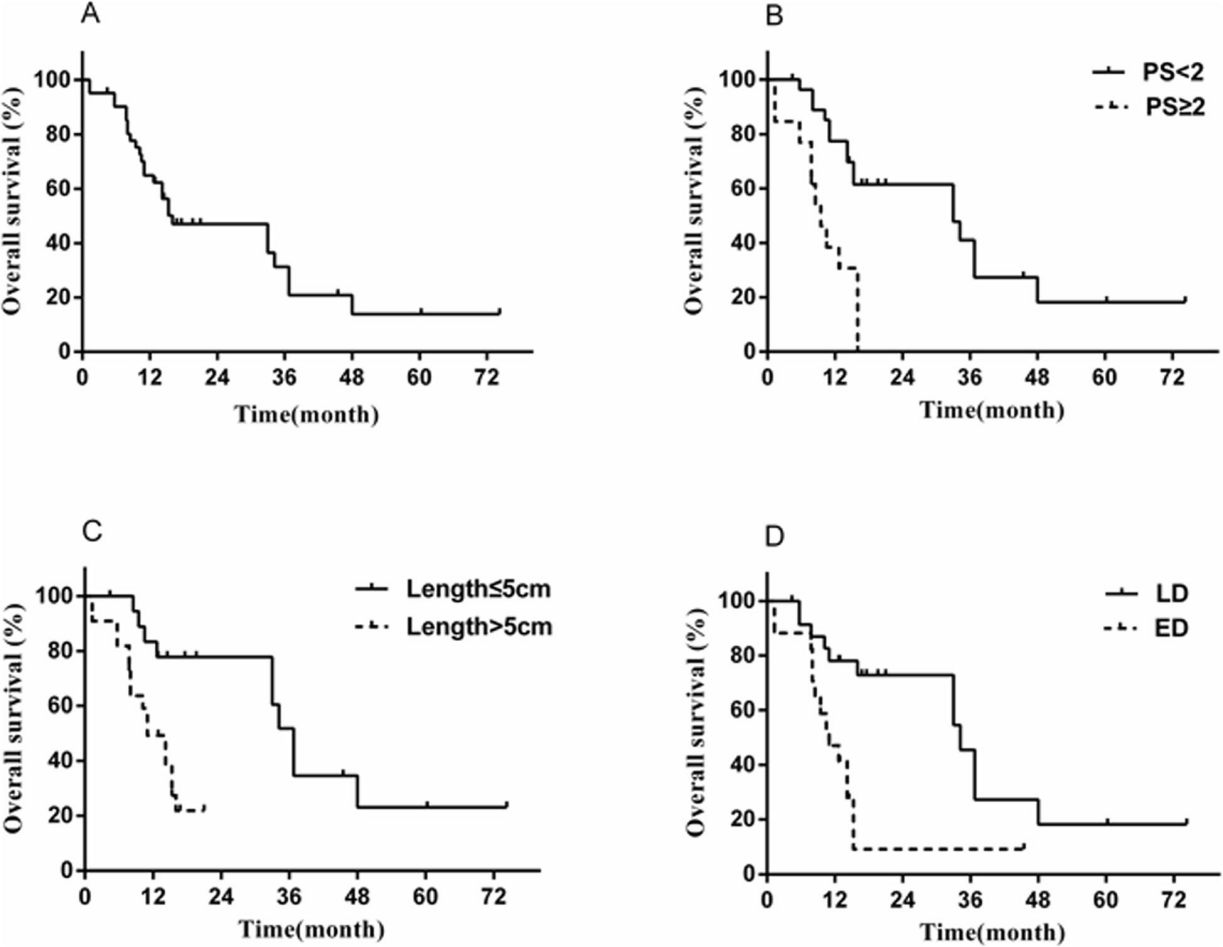
**a** Overall survival (OS) of all 42 patients; (**b**) OS of patients with ECOG PS of 0–1 or 2–3; (**c**) OS of patients with lesion length > 5 cm or ≤ 5 cm; (**d**) OS of patients with stage LD or ED

The OS rates at 1-, 3- and 5-years in the whole cohort were 64.9, 31.3, and 13.9%, respectively, with a median OS of 12.9 months (Fig. 1a). The OS rates at 1-, 3- and 5-years in the subgroup of patients with LD were 78, 45.5, and 18.2%, respectively, with a median OS of 36.8 months, whereas the 1-year and 3-years OS rates for ED patients decreased dramatically to 47.1 and 9.2%, accompanied by decrease in median OS to 11.0 months (Fig. 1d).

At the time of last observation, 16 patients survived with or without the primary disease, while 26 patients died of tumor recurrence or metastases. In patients with LD, four patients died of local recurrence disease, and eight patients died of metastases disease. Similarly, in patients with ED, five patients died of local recurrence disease and nine patients died of metastases disease. In total, 65% (17/26) patients died with the metastasis with or without the primary recurrence.

**Fig. 2:**
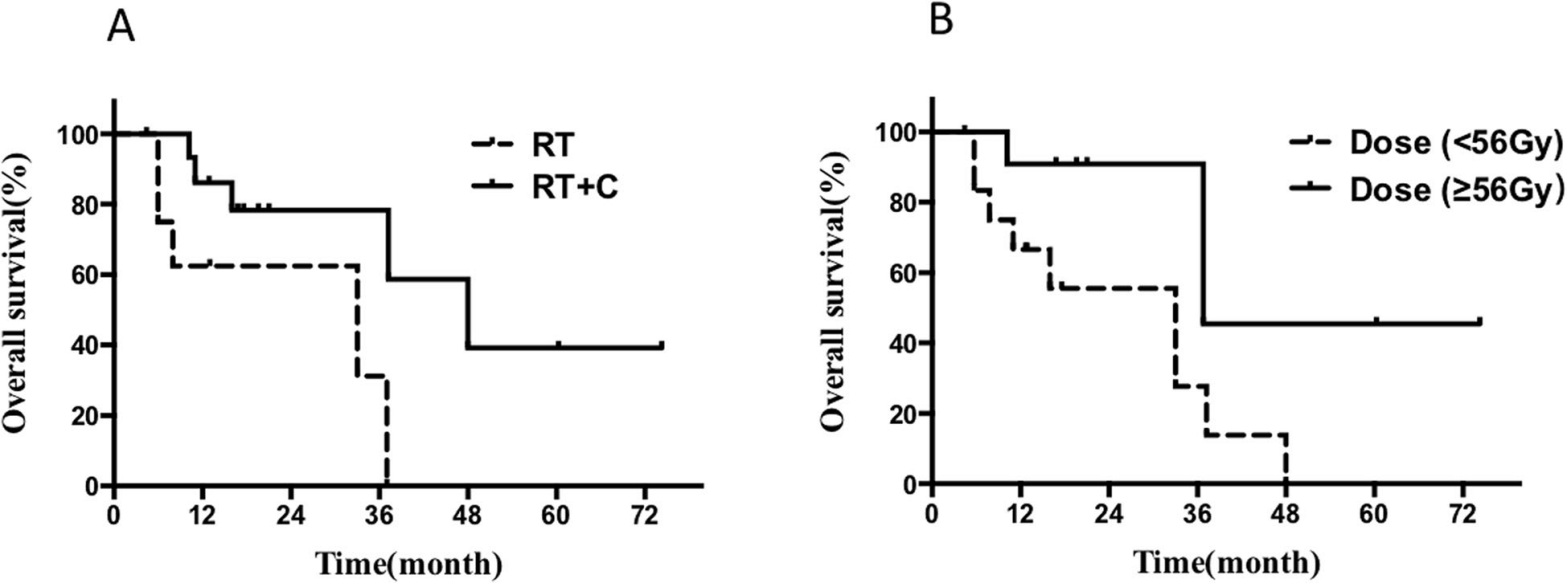
**a** OS of the patients with LD stage treated with RT vs RT + C; (**b**) OS of patients with LD stage treated with lower (<56Gy) vs higher dose(≥56Gy) of RT

The prognostic factors of the whole cohort and the patients with LD are shown in Table 3. ECOG PS, tumor length, stage, treatment modality (RT vs RT + chemotherapy), RT technology, and radiation dose were included into the Cox multivariate regression model as these variables were significant (*p* < 0.05) in the univariate analysis. Multivariate analysis suggested only ECOG PS, tumor length, and stage are the independent predictors for overall survival in the whole cohort (Fig. 1b-c). Regarding of the patients with LD, multivariate analysis indicated that combined with chemotherapy (HR = 0.204, 95%[CI] = 0.050–0.839, *P* = 0.046) and a higher radiation dose (HR = 4.212, 95%[CI] = 1.024–17.335, *P* = 0.027) predicted better prognosis in OS (Fig. 2a-b).

**Table 3:** Univariate analysis and multivariate Cox regression for prognosis of 42 SCCE patients

Factors	All cohort(*n* = 42)			LD(*n* = 25)		
	*P* value (Univariate)	*P* value (Multivariate)	HR (95% CI)	*P* value (Univariate)	*P* value (Multivariate)	HR (95% CI)
Age (< 60 years vs. ≥60 years)	0.280			0.989		
Sex (Male vs. Female)	0.777			0.271		
ECOG PS (≥2 vs. < 2)	0.000	0.001	6.314 (2.027–19.669)	0.048	0.291	2.577 (0.444–14.947)
Lesion length (> 5 cm vs. ≤5 cm)	0.001	0.001	8.593 (2.449–30.156)	0.19		
Smoking history (Yes vs. No)	0.578			0.212		
Risky diet (Yes vs. No)	0.731			0.731		
Stage (ED vs. LD)	0.002	0.049	2.786 (1.002–7.741)	–	–	–
Treatment (R + C vs. R Only)	0.003	0.088	0.302 (0.076–1.197)	0.028	0.046	0.204 (0.050–0.839)
Technology (3DCRT vs. IMRT)	0.039	0.941	0.948 (0.226–3.974)	0.498		
RT dose (<56Gy vs. ≥56Gy)	0.024	0.577	1.373 (0.451–4.184)	0.047	0.027	4.212 (1.024–17.335)

Abbreviations: *LD*: limited-disease, *ED*: extensive-disease, *RT*: Radiotherapy, *CT*: Chemotherapy, *HR*: Hazard Ratio, *CI*: Confidence Interval

### Adverse events

Most treatment-related toxicities were tolerable and reversible and of grade 1 to 2. Of the 24 patients who received radiation combined with chemotherapy, grade 3–4 leukocytopenia occurred in five (20.8%) patients (four with LD and one patient with ED). Other grade 3–4 toxicity included thrombocytopenia (*n* = 1), esophagitis (*n* = 1), and nausea and vomiting (*n* = 2). The overall rate of grade 3–4 toxicities was 37.5%. Toxicity was lower in 15 patients who received only radiation, of which four (25%) experienced grade 3–4 toxicity including leukocytopenia (*n* = 1), esophagitis (*n* = 2), and nausea or vomiting (*n* = 1). There was no radiation-induced lung injury or treatment-related death.

## Discussion

Several studies have shown that SCCE only accounts for 0.4–2.8% of primary esophageal carcinoma [6, 7]. Reports of increasing incidence of SCCE have come in from East Asian countries, including China and Japan, reaching global trends [8, 9]. Indeed, a comparison with our previous findings in 2012, the incidence rate of SCCE in our center rose slightly from 0.94 to 1.15%, perhaps due to the improvement in diagnostic pathology [5]. One study described discrepancies in tumor characteristics and therapeutic modalities in different ethnic groups [2]. For instance, the most common primary location of SCCE was the lower thoracic esophagus in the U. S population, while the Chinese population tends to have middle thoracic SCCE. Localized treatment modalities also differ between these two countries. Esophagectomy was the most popularized local therapy in China, which was performed in ~ 52 to 85% of patients [10, 11], whereas approximately half of the U.S. patients were reported to receive RT as the principal local therapy, regardless of chemotherapy [2]. Nevertheless, more Asian patients were diagnosed with earlier localized disease and the lack of feasibility of RT during the last few decades might explain this difference. We previously reported that 26.7% of patients in our center received RT as one part of the treatment, which increased to 40.9% in the current study, suggesting that RT combined with or without chemotherapy gained more popularity among treating physicians for SCCE recently [5].

The role of RT on the local control of SCCE is not well-illustrated because of the lacking of the information about the response rate in previous reports. In our study, the overall response (CR + PR) rate of 60% was obtained, suggested that RT alone or combined with chemotherapy was an effective option for locoregional disease control. Also, most published studies focus only on OS, but not PFS or DFS, leading to difficulty in evaluating the efficiency of RT in long-term local control. Our result indicated that the high response rate achieved by RT contributes to the survival benefit through long-duration remission. The median OS was 12.9 months in this study which was similar to previous reports (8–16 months) [2, 4]. The reported OS rates at 1-, 3- and 5-years in SCCE patients who received multiple therapeutics varied from 30 to 74.8%, 13.2–38.8%, and 7.8–18% respectively, and the survival data obtained in our study are almost equivalent to the best outcome [10–13).

As the two main local therapeutic modalities, surgery and definitive RT, especially for LD SCCE, show vast discrepancies when conducted in individuals with different demographics and clinical characteristics. While Sun et al.*,* retrospectively reported 1-, 3- and 5-year OS rates of 50.7, 13.7 and 8.2% in 73 SCCE patients treated by surgery, these rates are lower than findings we reported previously and here [14]. However, in an analysis of the National Cancer Data Base, esophagectomy was associated with the best OS for patients with localized or local-advanced SCCE comparing to chemoradiation or chemotherapy alone. The 1-, 3- and 5-year OS rates reported in another study were higher than those obtained in our study, but the median overall OS of LD-SCCE patients who received surgery was only 18.0 months, which was consistent with other report but shorter than current analysis [15, 16]. Interestingly, another study comparing the RT and chemotherapy (RT + CT) with surgery and chemotherapy (S + CT) in the management of LD SCCE indirectly favored RT, in which a significantly longer OS with RT + CT compared to S + CT (33.0 vs. 17.5 months, *p* = 0.02) was observed [13]. To sum up, there is a lack of consensus on whether surgery or RT should be the localized treatment modality for SCCE, especially for LD-SCCE, which must be further investigated in prospective controlled studies.

Here we also have shown that RT is also beneficial for the survival of patients with ED SCCE. Similar to the result of the current study, one accumulative analysis of the SEER data demonstrated that the addition of RT leads to a reduction of 30% risks of death in distant stage ESCC. Despite these promising results, the survival benefit of SCCE from RT was much poorer than that from squamous carcinoma or adenocarcinoma of the esophagus, suggesting the need for novel treatment modalities strategy for SCCE [17, 18]. Chemotherapy is one of the most important modalities for SCCE, it was well-acknowledged as the fundamental part of the multimodalities therapy and improved the survival both in LD and ED patients [13, 19]. In our study, the survival of RT + CT group prone to be longer compared to RT alone but without statistically significant difference (*p* = 0.088) of the whole cohort. In patients with LD, RT + CT is correlated with better survival (*p* = 0.046). By summary, the combination of RT and chemotherapy will further improve the survival of SCCE patients. Distant metastasis is the major failure pattern of SCCE despite stages. In accordance with this, over half (65%) of patients were failed in the distant control of our study, which further suggested that systemic chemotherapy is essential for the long-term control of SCCE.

The principles of chemotherapy for SCCE is poorly illustrated. According to a study focusing on the SCCE in locoregional disease treated with RT ± chemotherapy, more cycles of chemotherapy improved the survival, while the regiment and sequence of RT and chemotherapy had no impact on the disease control [4]. Unfortunately, due to a relatively small size of patients received chemotherapy in our study, we could not yield a reliable analysis to answer these questions. There is no consensus on the practice of RT neither, especially the technology and dose in SCCE, which are based on routine RT for SCLC. The dose used in this study is similar to the common RT doses ranging from 40-70Gy based on previous case reports and retrospective studies [20–22]. We found that a higher dose of RT (≥56Gy) is correlated with better survival. However, Jeene et al. reported that no effect of radiotherapy dose on overall survival in the multivariable analysis, while as it was mentioned, the results should have to be interpreted with caution, as only 5 patients received a higher dose of RT in that study. Despite knowing that IMRT is better at target positioning and avoiding errors for esophageal cancer compared to conventional RT [23, 24]. There are no comparative studies of these two RT technologies for SCCE treatment. We did not find significant differences between the OS of patients treated with 3DCRT or IMRT, which might be related to the small sample size of this study.

The main shortcomings of the current study are the small sample size and its retrospective nature. Nevertheless, compared to other studies, we only enrolled the patients who received RT as one part of the treatments and addressed on the local-control role of RT in SCCE by using consistent detailed individual patient data from a single institution. However, the confounding effect caused by enrolling patients with other treatment strategies cannot be overlooked.

Due to its low incidence, prospective and randomized studies on exploring the novel treatments for SCCE is limited. A phase II study (NCT03811379) in our center was initiated in 2018 and is currently recruiting patients to explore the efficacy of toripalimab (PD-1 antibody) as a monotherapy for patients with SCCE, which provides a hint for the novel exploration of a combination of immunotherapy and radiation therapy for SCCE.

## Conclusion

In summary, RT was one of the effective and safe treatment options for locoregional control of SCCE. Lower ECOG PS score, shorter lesion length, treated with chemotherapy, and a higher dose of RT were identified as favorable independent prognostic factors.

## Data Availability

The datasets used and/or analysed during the current study are available from the corresponding author on reasonable request.

## Abbreviations

3D-CRT: 3-Dimensional Conformal Radiotherapy
CR: Complete remission
CTV: Clinical target volume
ECOG PS: The Eastern Cooperative Oncology Group Performance Status Scale
ED: Extensive disease
GTV: Gross tumor volume
IMRT: Intensity Modulation Radiated Therapy
LD: Limited Disease
OS: Overall survival
PD: Progressive disease
PR: Partial remission
PTV: Planning target volume
RECIST 1.1: Response Evaluation Criteria in Solid Tumors 1.1
RT: Radiation Therapy
SCC: Squamous cell carcinoma
SCCE: Small cell carcinoma of the esophagus
SD: Stable disease
